# Nanoparticulate Impurities in Pharmaceutical-Grade Sugars and their Interference with Light Scattering-Based Analysis of Protein Formulations

**DOI:** 10.1007/s11095-015-1634-1

**Published:** 2015-01-30

**Authors:** Daniel Weinbuch, Jason K. Cheung, Jurgen Ketelaars, Vasco Filipe, Andrea Hawe, John den Engelsman, Wim Jiskoot

**Affiliations:** 1 Coriolis Pharma, Am Klopferspitz 19, 82152 Martinsried-Munich, Germany; 2 Division of Drug Delivery Technology, Leiden Academic Centre for Drug Research, Leiden University, PO Box 9502, 2300 RA Leiden, The Netherlands; 3 Sterile Product and Analytical Development, Merck Research Laboratories, Kenilworth, New Jersey USA; 4 Analytical Development and Validation, Biologics Manufacturing Sciences and Commercialisation, Merck Manufacturing Division, MSD, 5342 CC Oss, The Netherlands; 5 Analytical Department, Adocia, 69003 Lyon, France

**Keywords:** Dynamic light scattering, Excipients, Impurities, Nanoparticle tracking analysis, Protein formulation, Sucrose, Sugars

## Abstract

**Purpose:**

In the present study we investigated the root-cause of an interference signal (100–200 nm) of sugar-containing solutions in dynamic light scattering (DLS) and nanoparticle tracking analysis (NTA) and its consequences for the analysis of particles in biopharmaceutical drug products.

**Methods:**

Different sugars as well as sucrose of various purity grades, suppliers and lots were analyzed by DLS and NTA before and (only for sucrose) after treatment by ultrafiltration and diafiltration. Furthermore, Fourier transform infrared (FTIR) microscopy, scanning electron microscopy coupled energy-dispersive X-ray spectroscopy (SEM-EDX), and fluorescence spectroscopy were employed.

**Results:**

The intensity of the interference signal differed between sugar types, sucrose of various purity grades, suppliers, and batches of the same supplier. The interference signal could be successfully eliminated from a sucrose solution by ultrafiltration (0.02 μm pore size). Nanoparticles, apparently composed of dextrans, ash components and aromatic colorants that were not completely removed during the sugar refinement process, were found responsible for the interference and were successfully purified from sucrose solutions.

**Conclusions:**

The interference signal of sugar-containing solutions in DLS and NTA is due to the presence of nanoparticulate impurities. The nanoparticles present in sucrose were identified as agglomerates of various impurities originating from raw materials.

**Electronic supplementary material:**

The online version of this article (doi:10.1007/s11095-015-1634-1) contains supplementary material, which is available to authorized users.

## Introduction

The safety and efficacy of a therapeutic protein depends in part on its chemical and physical stability. Degradation, such as aggregation, of a therapeutic protein can reduce the availability of the protein’s active form, can negatively affect its pharmacokinetic properties and might cause adverse effects, such as unwanted immunogenicity [1–3]. To enhance the chemical and physical stability of a protein therapeutic, biopharmaceutical drug products contain a combination of specific formulation additives to ensure the chemical and physical stability of the therapeutic protein.

Among the many known excipients sugars, in particular sucrose and trehalose are employed, because they are preferentially excluded from the protein’s surface, thus, increasing the free energy of the system and thereby promoting conformational stability [4–6]. Examples of sugar-containing products on the market are amongst others Enbrel®, Avastin® and Stelara®. Sugars are also extensively used for lyophilized protein formulations as cryoprotectors and lyoprotectors, e.g., Herceptin®, Serostim® and Remicade [7]. As with all reagents that are approved for the use in pharmaceutical drug products, testing procedures and purity criteria of sugars are defined and regulated by the respective pharmacopeias.

Throughout the development of a therapeutic protein and its respective drug product, particle analysis is performed to assess product quality and protein stability. This practice has received increasing attention during the past few years and dynamic light scattering (DLS) became a commonly applied tool for this task in various phases of development, e.g., formulation screening, real-time or accelerated stability studies, and forced degradation studies. The value of DLS analysis comes from its wide size range it covers (from about a nanometer to several micrometers), the fast and easy performance, and its high sensitivity towards larger species, such as protein aggregates and particles [8, 9]. Despite its advantages, however, the analysis can be disturbed by the presence of certain excipients, which scatter light in the relevant size range, such as polysorbate micelles or sugar molecules. Sugar molecules have, according to the literature, a size of about 0.5 and 1 nm for mono- and disaccharides, respectively [10]. Interestingly, however, a second signal appearing at around 100–200 nm was consistently found when sugar-containing formulations were analyzed by DLS. In 2007, Kaszuba *et al*. explained the presence of this second signal as to be “probably due to collective diffusion of the sucrose molecules” [11]. Ever since, academic and industrial researchers have referred to this signal as the intrinsic phenomenon of sugar interference with DLS. Importantly, this interference marks a big challenge for DLS when analyzing biopharmaceutical drug products, because of difficulties in assessing the formation of aggregates and particles in presence of a permanent signal at 100–200 nm. It further impairs the ability to compare the stability of a protein formulated with different sugars or varying sugar content, e.g., during formulation development. Surprisingly and despite all these issues, the origin of this interference was never truly investigated.

Therefore, the present study was designed to understand the root-cause of the sugar interference with DLS, and its consequences for the analysis of particles in biopharmaceutical drug products. While all tested sugars (sucrose, trehalose, fructose, maltose and galactose) exhibit an interference phenomenon, we show on the example of sucrose that the interference is caused by the presence of actual nanoparticles, which dramatically differ in amount, but less so in size, between suppliers and between batches of the same supplier. A detailed characterization of these particles identified them as impurities originating from raw materials that are not completely removed during the refinement process. The quantities of nanoparticles present in pharmaceutical-grade sucrose were found to be up to 10^9^ particles per gram, while the product still can fulfill all requirements set by the current U.S. and European pharmacopeias.

## Materials & Methods

### Materials

Lysozyme was purchased from Fluka (Buchs, Germany), and a humanized monoclonal antibody, isotype IgG1 [12], was used to model a therapeutic protein. Sucrose was purchased from Sigma (Taufkirchen, Germany), Merck (Darmstadt, Germany), Caelo (Hilden, Germany), VWR (Bruchsal, Germany) and donated by Südzucker (Mannheim, Germany). PVDF syringe filters with a pore size of 0.2 and 0.1 μm were obtained from Millipore (Schwalback, Germany), Anotop syringe filters with a pore size of 0.02 μm were obtained from GE Life Science (Freiburg, Germany).

### Sample preparation

All saccharides were dissolved in Milli-Q® water (Millipore) at stated concentrations in percent weight per volume (% w/v). Protein (IgG or lysozyme) was dissolved in a 7% sucrose solution to achieve the desired concentrations. If not stated differently, all solutions were filtered through a 0.2-μm PVDF syringe filter.

### Diafiltration

A Minimate II Tangential Flow Filtration (TFF) system (Pall, Crailsheim, Germany) equipped with a 30 kDa TFF capsule (Pall) was used to perform diafiltration on 700 mL of an aqueous sucrose G solution (50% w/v). Diafiltration against Milli-Q® water was performed until the permeate volume reached 14 times the feed volume. The last filtrate volume was analyzed by DLS and did not show any residual sucrose peaks. The residual sucrose monomer concentration after diafiltration (c_DF_) was calculated as 0.3 mg/L, according to Eq. (1):

1 $$ {c}_{DF}={c}_I\cdot {e}^{-N} $$

where c_I_ is the initial sucrose monomer concentration, N the number of diavolumes, and where no retention of the sucrose monomer by the TFF membrane is assumed. Subsequently, the retenate was concentrated by first using TFF and then 10-kDa centrifugal filter-units (Amicon Ultra 15, Millipore) to a final volume of ca. 0.8 mL. As a control, Milli-Q® water without the addition of sucrose was treated the same way.

### Dynamic Light Scattering (DLS)

DLS measurements were performed with a Zetasizer Nano ZS system (Malvern, Herrenberg, Germany) equipped with a 633 nm He-Ne laser. The scattered light was detected by using non-invasive backscatter detection at an angle of 173°. A sample volume of 500 μL was analyzed in single-use polystyrene semi-micro cuvettes with a path length of 10 mm (Brand, Wertheim, Germany). The Dispersion Technology Software version 6.01 was used for data collection and analysis. If not stated differently, the measurements were made with an automatic attenuator and a controlled temperature of 25°C. The intensity size distribution, Z-average diameter, derived count rate, and polydispersity index were calculated from the autocorrelation function obtained in ’general purpose mode’. Each sample was measured in triplicate.

### Nanoparticle Tracking Analysis (NTA)

NTA was performed with a NanoSight LM20 (NanoSight, Amesbury, UK). The instrument was equipped with a 405 nm blue laser, a sample chamber and a Viton fluoroelastomer O-ring. If sample dilution was necessary to achieve an optimal concentration for NTA, Milli-Q® water was used as a diluent and all results were calculated back to the original concentration. Samples were loaded into the sample chamber by using a 1-mL syringe and a pre-run volume of 0.5 mL. Samples were analyzed in triplicate at a stopped flow, while 0.1 mL was flushed through the chamber between each repetition. The NTA 2.3 software was used for capturing and analyzing the data. Movements of the particles in the samples were recorded as videos for 60 s, while the shutter and gain settings of the camera were set automatically by the software for an optimal particle resolution.

### UV-spectroscopy

UV-spectroscopy was performed in UV-transparent 96-well plates (Corning Incorporation, NY, USA) by using a Tecan Safire^2^ plate reader (Tecan Austria GmbH, Grödig, Austria). For each data point, 200 μL of sample was measured in triplicate, each measurement being an average of 20 reads.

### Fluorescence Spectroscopy

Fluorescence spectroscopy was performed in black 96-well plates (Corning Incorporation, NY, USA) by using a Tecan Safire^2^ plate reader (Tecan Austria GmbH, Grödig, Austria). Excitation and emission of a 200-μL sample were 3D-scanned in triplicate, each measurement being an average of 20 reads from 250 to 460 and 290 to 600 nm, respectively.

### Scanning Electron Microscopy Coupled Energy-Dispersive X-ray Spectroscopy (SEM-EDX)

SEM-EDX measurements were performed with a Jeol JSM-6500F instrument (Jeol, Tokyo, Japan) equipped with a silicon drift detector (Oxford Instruments, Abingdon, U.K.). For preparation 90 μL of each sample was dried under vacuum and at room temperature on top of a sterile plastic coverslip (Nunc Thermo Scientific, Schwerte, Germany), which was fixed onto a SEM-sample holder with an electrically conducting double-sided tape (Plano, Wetzlar, Germany). A self-sticking copper band (Plano) was used to electrically connect the sample surface to the sample holder base. The sample surface was then carbon-coated by using a Bal-Tec MED-020 carbon evaporator (Bal-Tec, Wetzlar, Germany).

### Fourier Transform Infrared Microscopy (FTIR)

FTIR measurements were performed on dried samples with a Bruker Hyperion 3000 FTIR microscope equipped with an attenuated total reflection (ATR) objective (Bruker Optics, Ettlingen, Germany) operated by the Bruker Opus 6.5 software. Samples were dried and prepared as described for SEM-EDX analysis, but without the application of a copper band and without carbon coating.

## Results

**Fig. 1:**
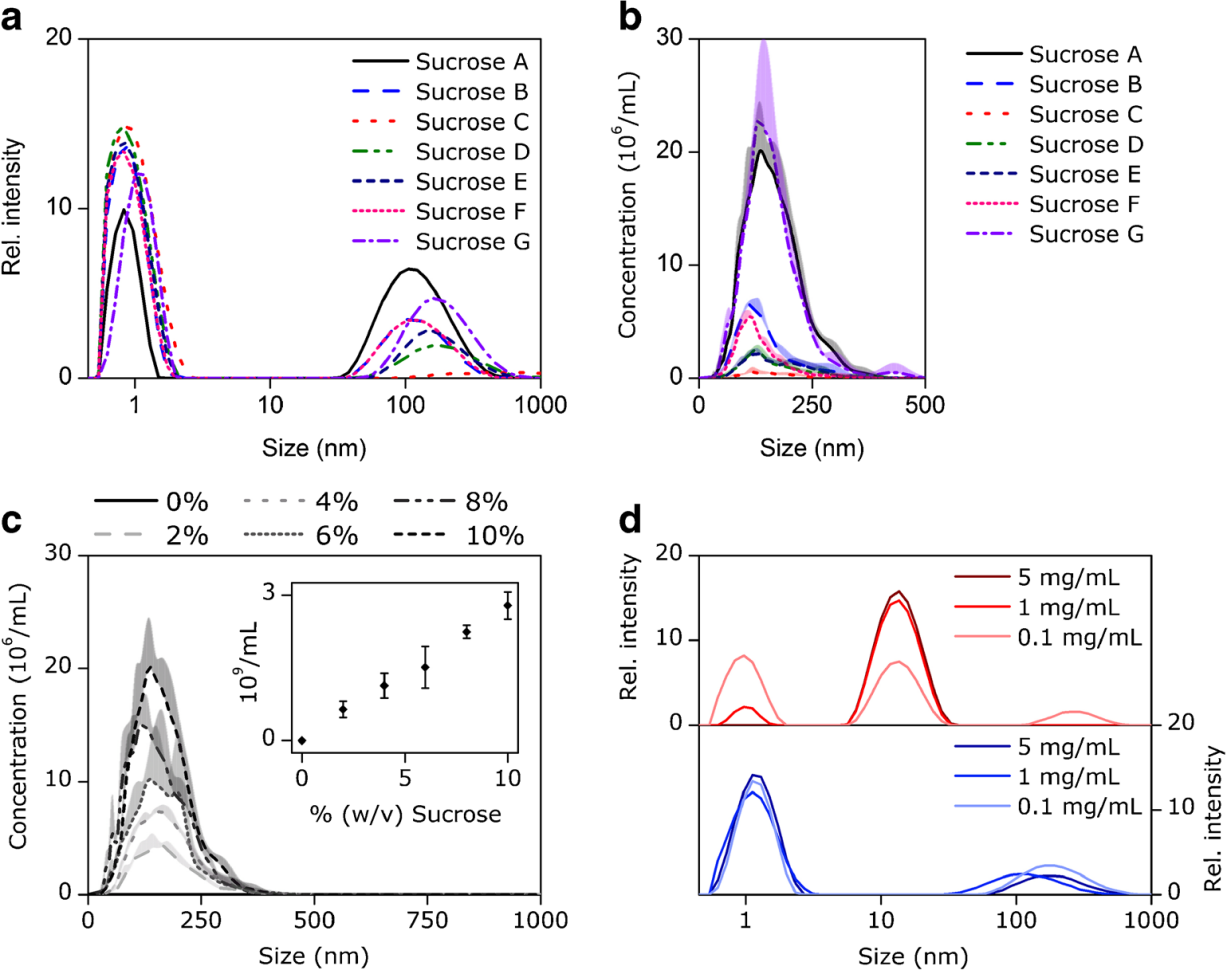
(**a**) Intensity-weighted size distribution by DLS and (**b**) particle size distribution by NTA obtained for different sugars in aqueous solution at 10%. (**c**) Total particle concentration (insert) and particle size distribution obtained by NTA for sucrose A solution from 0 to 10%. (**d**) Intensity-weighted size distribution by DLS for 7% sucrose A solutions containing increasing concentrations of IgG (*upper panel*) and lysozyme (*lower panel*). Shown are mean values (**a**-**d**) plus standard deviations (**b** and **d**) obtained from triplicate measurements.

Various sucrose products (Table I) were analyzed as 10% solutions by DLS and all showed two distinct peaks in the intensity-weighted size distribution (Fig. 1a). The position of the first peak correlates to the literature value for the hydrodynamic diameter of a sucrose molecule in water of 0.98 nm [10]. The second peak showed its intensity maximum at ca. 100 to 200 nm for all samples except sucrose C, for which the peak appeared at about 1900 nm. The relative intensity area under the curve (AUC) of this signal varied considerably between samples, ranging from 8.3% for sucrose C to 60.3% for sucrose A, while differences were observed between purity grades, suppliers, and also between batches of the same supplier (Table I). Also in NTA, a signal at about 100–200 nm was detected with little variation in size distribution but high variations in particle concentration between products (Fig. 1b, Table I). Furthermore, an increase in concentration of sucrose A in water resulted in a linear increase in nanoparticle concentration determined by NTA, while a water control did not show any particles (Fig. 1c). Furthermore, the size distribution did not change with increasing sucrose concentration. Additionally, triplicate sample preparations analyzed by DLS and NTA showed high repeatability (data not shown).

**Table I:** Sucrose products used in this study and DLS and NTA results of (10% w/v) sucrose in solution. Numbers show mean values of triplicate measurements

	Supplier	Grade	Lot	DLS					NTA			
				Z-Average (d. nm)	PDI^c^	Derived count rate	Peak 1 (nm)	Peak 2 (nm)	Concentration (10^8^/mL)	D10 (nm)	D50 (nm)	D90 (nm)
Sucrose A	Sigma	ACS^a^	SLBD1571V	13.7	0.95	247	0.9	133	27.9	94	158	246
Sucrose B	Sigma	Ph.Eur.^b^	SZBC012V	4.3	0.35	160	0.9	134	7.1	82	131	238
Sucrose C	Merck	Ph.Eur.	K42570987144	1.3	0.12	147	1.0	1899	0.7	96	160	312
Sucrose D	Merck	Ph.Eur.	K38684287934	2.4	0.20	151	0.9	216	2.8	91	147	276
Sucrose E	Südzucker	Ph.Eur.	L115310600	15.4	0.24	161	0.9	188	3.0	95	161	267
Sucrose F	Caelo	Ph.Eur.	12241808	4.2	0.34	157	0.9	139	4.9	81	122	206
Sucrose G	VWR	Ph.Eur.	13C190006	10.0	0.58	182	1.1	202	27.9	94	153	237

^a^Purity meets or exceeds the standards of the American Chemical Society ^b^Purity meets or exceeds the requirements of the current European Pharmacopeia ^c^Polydispersity index

IgG and lysozyme formulated at various concentrations in 7% sucrose A solutions were analyzed by DLS. At an IgG concentration of 0.1 mg/mL, the signal from the sucrose molecule (1 nm), the IgG (14 nm) and the 100–200 nm signal were visible (Fig. 1d, upper panel). At 1 mg/mL, the 100–200 nm signal disappeared and at 5 mg/mL also the sucrose signal (1 nm) vanished, leaving only the signal from the IgG. For lysozyme (Fig. 1d, lower panel), the 100–200 nm signal was detected in presence of all tested protein concentrations (0.1–5 mg/mL), while the signal of the sucrose molecule and lysozyme likely overlapped at about 1–2 nm because of the poor resolution of DLS [13].

**Fig. 2:**
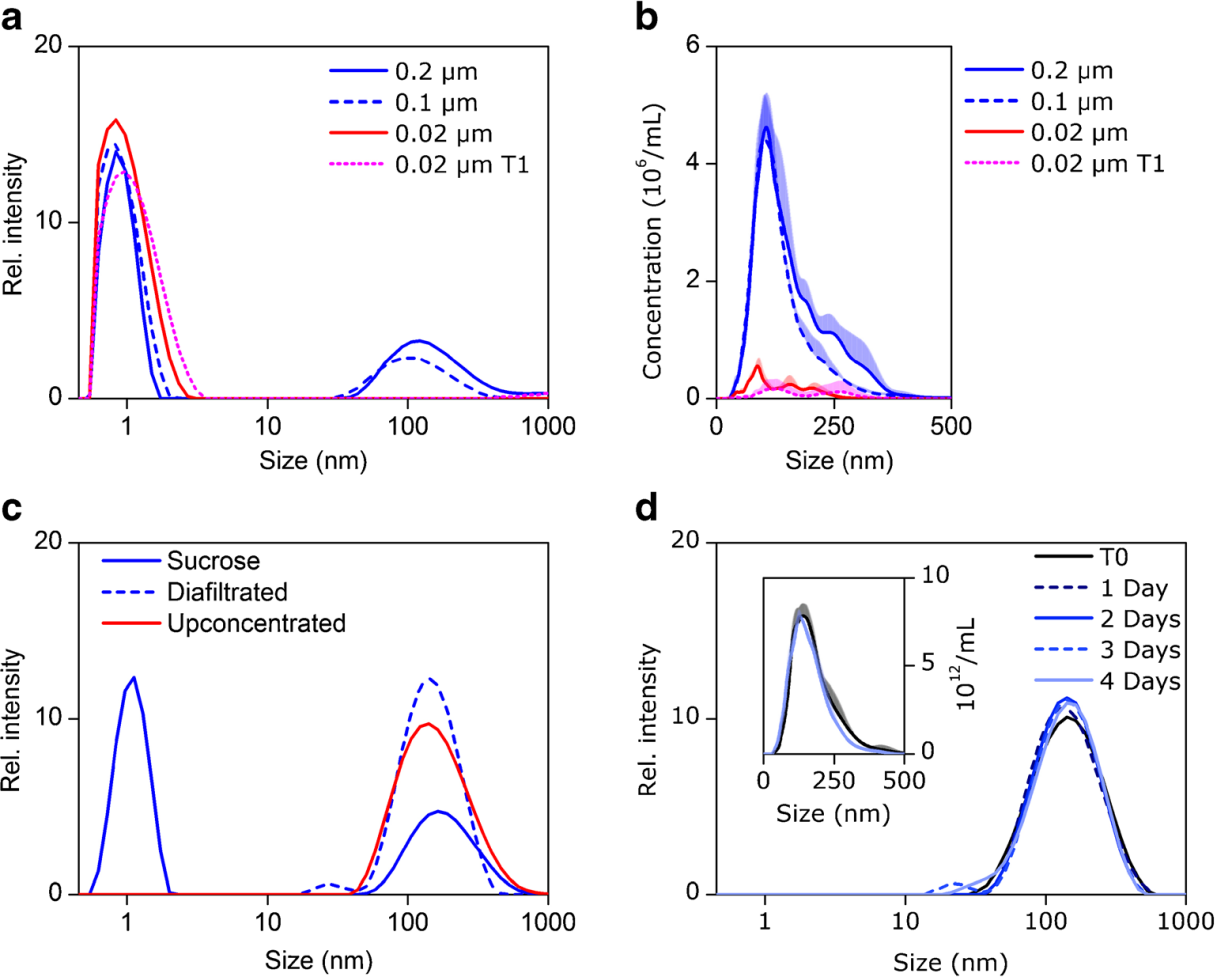
(**a**) Intensity-weighted size distribution by DLS and (**b**) particle size distribution by NTA obtained for sucrose B solutions (10%) after filtration (stated pore size) and storage for 4 days at 25°C (T1). **c** Intensity-weighted size distribution of a 10% sucrose G solution before and after diafiltration and subsequent upconcentration as determined by DLS. D) Intensity-weighted size distribution by DLS and particle size distribution by NTA (insert) of a diafiltrated 10% sucrose G solution stored at 25°C.

Solutions of sucrose B were filtered through filters with decreasing pore size and subsequently analyzed by DLS and NTA (Fig. 2a and b). Filtering the solutions through a 0.1-μm filter had a small effect on the size, and little to no effect on the intensity of the 100–200 nm signal. However, filtration through a 0.02-μm filter decreased the signal in both DLS and NTA to background levels and the signal did not reappear after incubation of the filtered sample for 4 days at 25°C (T1). Moreover, it was possible to eliminate the signal from the sucrose monomer peak in a sucrose G solution by using diafiltration (Fig. 2c). The purified retentate (before concentrating) maintained a stable size distribution and nanoparticle concentration when incubated at 25°C for 4 days, as determined by DLS and NTA (Fig. 2d).

**Fig. 3:**
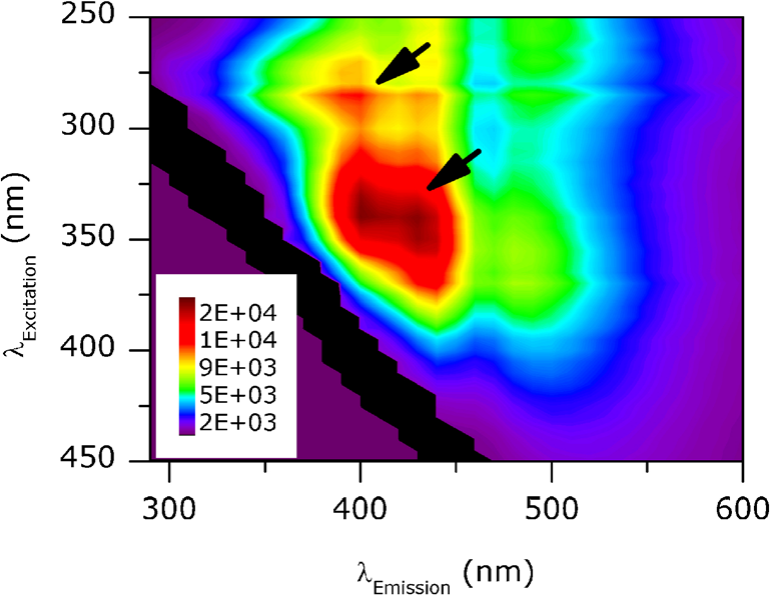
Fluorescence intensity landscape of suspended nanoparticles isolated from sucrose G. The arrows indicate areas of fluorescence maxima. The black area showed strong light scattering and was excluded from the analysis.

Upon concentration of the diafiltrated sucrose G retentate containing the nanoparticle fraction, the sample developed a brownish-yellow color and showed an increase in UV_420nm_ absorbance from 0.03 to 0.18 AU. A water control treated the same way as the sucrose G sample showed no particles by DLS and NTA and had an unchanged UV_420nm_ absorbance of 0.02 AU after concentration. Intrinsic fluorescence of the concentrated sample was analyzed to help identifying potential colorants. The fluorescence intensity landscape is shown in Fig. 3. Two distinct patterns of maximum fluorescence intensity could be identified in the sample, pattern 1 at ca. 280/390 nm (λ_Ex_/λ_Em_) and pattern two at ca. 340/420 nm. The water control treated equally did not show any intrinsic florescence (data not shown).

**Fig. 4:**
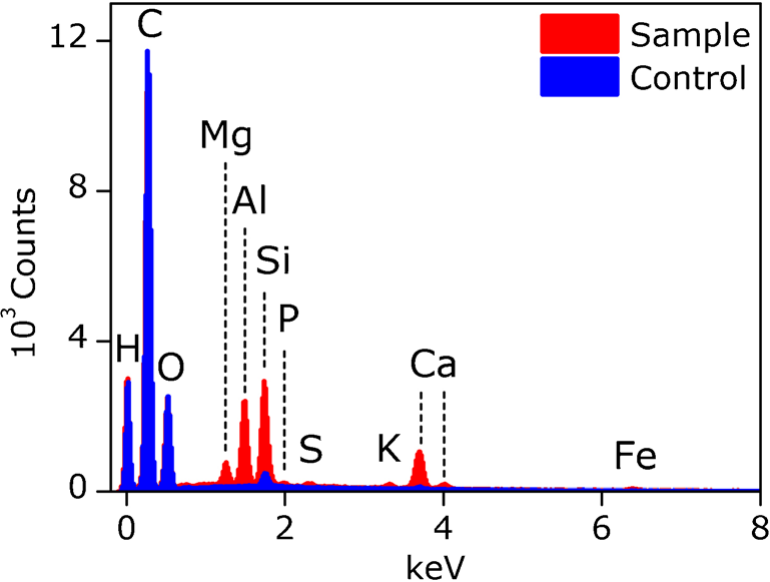
EDX spectrum of vacuum dried nanoparticle isolated from sucrose G (sample) against a water control treated the same way (control). Element analysis was performed against internal standards of the SEM-EDX system.

When the concentrated particle suspension, derived from sucrose G, was vacuum-dried, a thin and compact film layer formed, which did not show any particulate structures by SEM analysis. Rather, the film layer swelled and subsequently ruptured upon extended exposure to the SEM beam, suggesting water entrapment and thus potentially hygroscopic behavior (Figure S1). No particulate matter was visible by SEM on a vacuum-dried 0.02-μm filter after passing through the concentrated nanoparticle suspension (data not shown). Analysis of the film layer by EDX, however, revealed the presence of several minerals and metals. Signals from silicium, aluminum, calcium, and magnesium were detected, as well as small amounts of phosphor, sulfur, potassium, and iron (Fig. 4). The control sample, water processed equally, showed small amounts of silicium and calcium. Carbon, oxygen, and hydrogen signals were also detected, but are method derived and cannot be attributed to the sample.

**Fig. 5:**
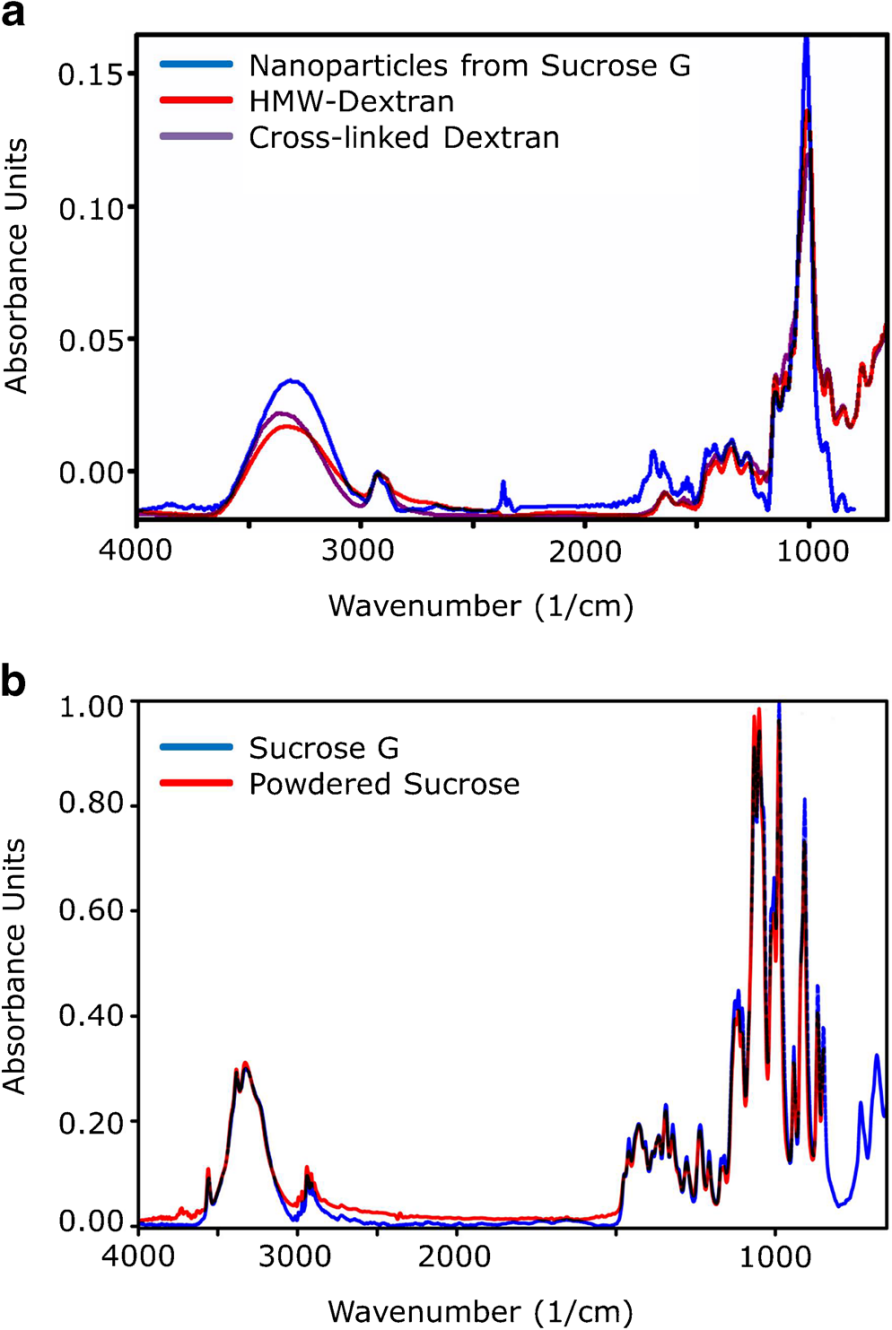
FTIR spectra recorded by FTIR microscopy overlaid with the best fitting entries of the S.T. Japan Europe GmbH database from 2009. (**a**) Recorded spectrum of vacuum dried nanoparticles isolated from sucrose G (*blue*) overlaid with the entries of high-molecular-weight (*red*) and cross-linked dextran (*violet*). (**b**) Recorded spectrum of unprocessed sucrose G (*blue*) overlaid with the entry of powdered sucrose (*red*).

FTIR microscopy was performed on the vacuum-dried sample to detect and identify potential organic material (Fig. 5). An FTIR spectrum was obtained that, when compared with the S.T. Japan-Europe GmbH library from 2009, matched closest the spectra of high-molecular-weight dextran (40 kDa, entry# 2130) and cross-linked dextran (Sephadex® G-50, 1.5–30 kDa, entry# 8096), with a hit quality of 626 and 620, respectively, with 1000 being a perfect match. Unprocessed sucrose G powder provided an FTIR spectrum that matched that of powdered sucrose (entry# 9772), with a hit quality of 959.

## Discussion

The interference of sugar-containing solutions with DLS analysis has been observed previously and manifests itself through an additional signal at ca. 100–200 nm, besides the signal at about 1 nm originating from the sugar monomer [11, 14]. In our study, we found this second signal in solutions of a variety of different sugars (trehalose, fructose, maltose and galactose, data not shown) and different sucrose products (Fig. 1a), confirming these previous observations. The 100–200 nm signal in DLS could mistakenly be interpreted as an aggregate peak and mask the formation/presence of protein aggregates. Although this signal will disappear at higher protein concentrations, it should be noted that several antibody drugs are formulated with a sugar at protein concentrations between 1 and 5 mg/mL [15], where the interference signal will likely show up (Fig. 1d). Moreover, blinatumomab, recently approved by the FDA, is formulated at a concentration as low as 12.5 μg/mL and several other protein therapeutics, such as epoetins [16] and cytokines [17], are formulated at similarly low concentrations. Furthermore, during early-stage formulation development, proteins are often used at low concentrations because of limited amounts of material available.

Up to now, the interference was suggested to be an intrinsic phenomenon coming from the sugar molecules themselves. However, if the 100–200 nm signal was indeed an intrinsic phenomenon caused by the sugar molecules, one would expect the interference to be the same for solutions of the same sugar concentration. In contrast, our results could demonstrate high variability of this interference for sucrose across purity grades, suppliers, and also across batches of the same supplier. Further, one batch supplied by Merck (sucrose C) showed this signal to a barely detectable, very low extent and the signal also deviated in size from that of the other products (Table I). Altogether, this indicates that the interference is caused by particulate matter rather than by monomeric sucrose molecules.

Besides DLS, also NTA detected particles at 100–200 nm showing high variability in particle concentration between the different sucrose products (Fig. 1b). Furthermore, the particle concentrations determined by NTA correlate, in relative terms, well with the polydispersity index and the derived count rate determined by DLS using a fixed attenuator (Table I). Thus, the particles detected by NTA are likely the same as those causing the signal in DLS. It should be noted that sucrose, lysozyme and IgG monomers are below the lower size limit of NTA [18]. However, they are detected by DLS, but their signal can in some cases, when a large protein such as an IgG is formulated at high concentration, decrease or even disappear in DLS analysis (Fig. 1d). Profound evidence that the presence of suspended particles is responsible for the interference signal comes from the results shown in Fig. 2a and b, where this signal in DLS and NTA disappeared after ultrafiltration (0.02 μm). The signal did not re-emerge from the remaining sucrose molecules in solution over the observed time frame of 4 days, suggesting an origin other than an intrinsic phenomenon of the sucrose molecules. After purification by diafiltration, the nanoparticles likely responsible for the interference did not dissolve or further agglomerate to larger particles, at least not readily, when stored in water, supporting the theory of the presence of stable and potentially foreign particulates (Fig. 2d).

Following the indication that the nanoparticles might be partially or fully composed of impurities or contaminants, a detailed chemical analysis of the nanoparticles was attempted. No particle like structures could be visualized by SEM analysis of a vacuum-dried particle suspension, because the sample preparation resulted in the formation of a film layer. However, the presence of inorganic elements was determined in this layer by the SEM coupled EDX analysis (Fig. 4). The combination of detected elements closely matches the description of an inorganic contaminant called ash, which is a combination of chlorides, sulfates, phosphates, silicates and minerals including calcium, potassium, magnesium and aluminum, mostly present as salts or oxides, as well as clay and sand [19]. Ash can enter the sugar cane or beet during growth from the soil, water and added fertilizers, but can also be introduced to the unprocessed sugar by external matter such as dirt or trash. Ash therefore commonly contaminates the unprocessed cane or beet juice, however, to various degrees and with slight differences in composition depending on the producer. Even though ash is largely cleared off by current refinement processes, an effective removal of ash components in refined white sugar products is still challenging for the sugar industry [20].

In the dried particle suspension, we could also detect dextran structures by ATR-FTIR microscopy (Fig. 5a). The data suggest that dextran is present as cross-linked fibers, likely responsible for the formation of the hygroscopic film layer upon drying the particles. Dextran is a well-known impurity in the sugar industry, produced due to enzymatic deterioration by *Leuconostoc* bacteria, which mainly enter the sugar cane or beet during harvesting, cutting and grinding, but can also be introduced in later production steps [21]. The dextran content in the unprocessed cane or beet juice, however, can vary significantly between different producers, depending amongst others on the delay time between cutting and milling, the harvesting method, the refinement process, and the overall hygiene [22]. Importantly, investigations have shown that dextran is not completely removed by current sugar refinement processes [23, 24].

It should be noted that both, ash and dextran, are essential components of molasses, a side product of sugar refinement giving brown sugar its distinct color. U.S. and European pharmacopeias require a color test and also UV absorbance data at 420 nm to specifically test pharmaceutical-grade sucrose for molasses remains. As described in the results section, we observed a brownish-yellow color and an increased UV absorbance at 420 nm after concentrating the nanoparticle impurities. The nanoparticle impurities further possessed fluorescence activity in two distinct regions (Fig. 3). Diverse amounts of fluorescent impurities of different compositions have been found in various sugar products by other research groups [20, 25–29]. According to these studies, the observed fluorescence patterns are caused by a combination of various fluorophores, two of which have close similarities with tryptophan and tyrosine and could be responsible for the fluorescence pattern one at ca. 280/390 nm [25–27]. Other fluorophores were identified as catechols formed by base-catalyzed sugar degradation and again other are suggested being Maillard reaction polymers, all of which could be potential contributors to the fluorescence pattern 2 [28, 29]. Fluorescent impurities can be found in various sugar products, however, in different compositions and quantities.

Dextran impurities found in sucrose occur in a wide molecular-weight-range from a few kDa to several MDa [21, 22], while the ash components detected by EDX and the components suggested by fluorescence spectroscopy are likely much smaller in size. Interestingly, all of those were found in the same particle population with a consistent size of 100–200 nm. Thus, two questions arise from there: i) How do the various impurities come together to form particles and ii) why do these particles occur in such a defined size distribution, even across various producers? A potential answer to these questions lies in the sugar refinery process itself, particularly in the carbonation or phosphatation step. Here, calcium carbonate or calcium phosphate, respectively, is formed, which co-precipitates with high-molecular-weight components and suspended solids [20]. During this step, agglomeration of dextran and other impurities and contaminants could lead to the formation of suspended nanometer sized particles. After the precipitation, the sugar juice is usually clarified by filtration where the membrane’s cutoff might be responsible for the defined size distribution of the nanoparticle impurities.

While the exact particle formation process is still rather speculative, it is worth discussing potential ways to deal with nanoparticle impurities in sugars. On the one hand, this could be attempted analytically. For measurements performed by DLS, however, it is not possible to mathematically calculate or subtract the contribution of the nanoparticle impurities from the signal. For measurements performed by NTA, a simple subtraction of the particle counts in the placebo buffer from the particle counts in the sample is possible. Nevertheless, it needs to be noted that the concentration of nanoparticle impurities at pharmaceutically relevant sucrose concentrations can exceed protein particle concentrations even in degraded samples by several orders of magnitude, making simple buffer subtraction statistically meaningless. On the other hand, a pharmaceutical manufacturer could get rid of the nanoparticles through the filtration of sucrose solutions using small pore size filters (e.g., 0.02-μm pores) with commonly available systems for production scale ultrafiltration. It would also be beneficial to improve the sugar refinement processes in order to reduce the amount of impurities in the final sugar product, as has been suggested by various research groups [19–23, 30]. To ensure effectiveness, however, it would then require monographs to include a test for nanoparticulate impurities in pharmaceutical-grade sugar products.

## Conclusions

In this study we demonstrated that sugar, even in pharmaceutical-grade quality, can contain up to 10^9^ nanoparticles per gram in the 100–200 nm range, which can limit the use of techniques for subvisible particle analysis, such as DLS and NTA. The number of nanoparticles can vary significantly between suppliers, as well as between production batches. This makes it very challenging to compare aggregation states of proteins in sugar-containing formulations by DLS and NTA, especially during formulation development. Our results indicate that the nanoparticles found in sucrose are agglomerates of a variety of impurities (dextrans, ash and aromatic colorants) that were not entirely removed during refinement processes. Importantly, the presence of these nanoparticulate impurities is not taken into consideration by pharmacopeial quality criteria. Furthermore, the nanoparticle impurities cannot be removed by common sterile filtration using a 0.22-μm pore size filter. However, ultrafiltration could be an effective way to clear the nanoparticles from sucrose solutions. Whether the particles observed in sugars other than sucrose are composed similarly and whether or not these impurities have an impact on a protein’s overall stability is currently unknown and is the subject of ongoing follow-up studies.

## Electronic supplementary material

Below is the link to the electronic supplementary material.

Figure S1 SEM image of vacuum dried nanoparticles isolated from sucrose G, showing a thin and compact film layer that ruptured under the heat of the SEM beam. (JPEG 46 kb)

## Abbreviations

ATR: Attenuated total reflection
AU: Absorbance units
AUC: Area under the curve
Da: Dalton
DLS: Dynamic light scattering
EDX: Energy-dispersive X-ray spectroscopy
FTIR: Fourier transform infrared spectroscopy
IgG: Immunoglobulin type G
NTA: Nanoparticle tracking analysis
PVDF: Polyvinylidene fluoride
SEM: Scanning electron microscopy
StDev: Standard deviation
UV: Ultra-violet
λ_Ex_/λ_Em_: Wavelength of excitation/emission
