# Supplementary figures and images for: Nanoparticulate Impurities in Pharmaceutical-Grade Sugars and their Interference with Light Scattering-Based Analysis of Protein Formulations
Source: Pharm Res. 2015 Jan 30;32(7):2419–27. doi: 10.1007/s11095-015-1634-1 (PMC4452213; doi:10.1007/s11095-015-1634-1)

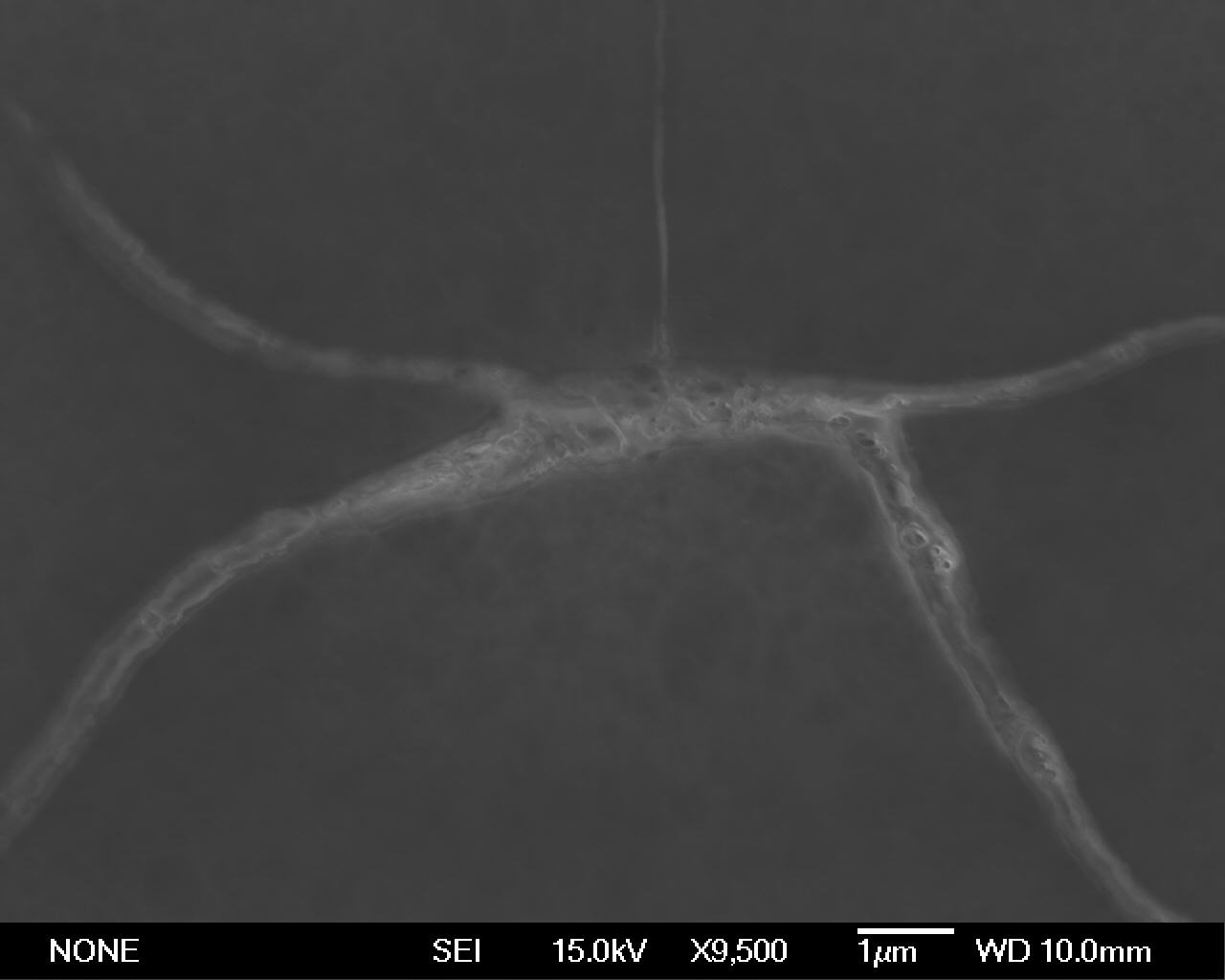

Supplement: Supplementary file 1 — SEM image of vacuum dried nanoparticles isolated from sucrose G, showing a thin and compact film layer that ruptured under the heat of the SEM beam. (JPEG 46 kb) [file 11095_2015_1634_MOESM1_ESM.jpg]
